# Association between sarcopenia and hearing impairment in middle-aged and elderly people in China: a prospective cohort study

**DOI:** 10.1038/s41598-024-56850-4

**Published:** 2024-03-13

**Authors:** Zeqi Zhang

**Affiliations:** Management School, University of Liverpool, Liverpool, L69 3BX UK

**Keywords:** Signs and symptoms, Health care, Geriatrics

## Abstract

This study used longitudinal data from CHARLS 2011–2018 for cross-sectional and longitudinal analyses to investigate the relationship between sarcopenia and hearing impairment in middle-aged and elderly adults in China. The study selected 9723 participants aged 45 years and older from CHARLS 2011 and followed up in 2015 and 2018. Binary logistic regression and cox proportional risk regression models were used for testing. The results of the study showed that in the cross-sectional analysis, probable sarcopenia was significantly associated with hearing impairment compared with the group without sarcopenia [OR (95% CI) 0.342 (1.187, 1.669), *p* < 0.001], but sarcopenia was not significantly associated with hearing impairment. In the longitudinal analysis, middle-aged and elderly adults with sarcopenia [HR (95% CI) 0.354 (1.043, 1.945), *p* < 0.01] were more likely to have hearing impairment than those with probable sarcopenia and without sarcopenia. Probable sarcopenia was strongly associated with hearing impairment in middle-aged and elderly adults, whereas sarcopenia was a strong predictor of hearing impairment over the next 7 years. The results of this study emphasize the urgent need for measures to address sarcopenia in order to prevent and delay the decline in hearing function.

## Introduction

The trend of global aging is intensifying, and China is no exception. As of 2022, there are 600 million people over the age of 45 in China, accounting for about 41.97% of the total population^1^. The rapid increase of the middle-aged and elderly has significantly influenced various aspects of healthcare, employment, and social construction, greatly increasing the social burden of elderly care and healthcare demands. In 2015, China’s expenditure on elderly care, medical treatment, nursing, and welfare accounted for 7.33% of GDP, and it is projected to increase to 26.24% by 2050^2^. With age, the physical functions of middle-aged and elderly individuals gradually decline, posing threats and hazards to their physical and mental health. Therefore, timely attention, diagnosis, and intervention in their physical health conditions are essential. It can not only enhance the health awareness of the middle-aged population, prevent and delay diseases and functional impairments, but also improve their quality of life and promote healthy aging.

Among the functional impairments of the middle-aged and elderly, hearing impairment is the most common^3^. Age-related hearing impairment (ARHI) is the decline in auditory function due to the aging of the auditory system^4^. WHO estimates that more than 1.5 billion people (one in five) worldwide have varying degrees of hearing impairment^5^. It is expected that by 2050, 2.5 billion people worldwide will have a hearing impairment^5^. According to data from China’s sixth population census, 30% of the population aged 60 years and older suffers from hearing impairment^6^. In 2020, the percentage of hearing impairment among people aged 65 years and older is already close to 50%^6^. Age-related hearing impairment is the leading cause of hearing disability in China^7^. Hearing impairment causes middle-aged and elderly people to be less sensitive to sound, which in turn affects their daily communication and cognitive abilities, hinders social participation and information exchange, and reduces the quality of life^3,8^. In addition, hearing impairment affects the physical activities of middle-aged and elderly people, leading to negative emotions such as loneliness, anxiety, and depression, and affecting mental health^3^. Previous studies have shown that hearing impairment in middle-aged and elderly adults is affected by genetic factors, smoking, occupational noise, and ototoxic drugs^9–11^. Recent studies have found that chronic diseases such as diabetes, cardiovascular disease, and metabolic syndrome also affect hearing function in middle-aged and elderly adults^12–15^. Among them, high BMI and high waist circumference are considered important risk factors for hearing impairment^11,16^. The pathophysiologic mechanisms of sarcopenia and metabolic syndrome are similar. Therefore, it can be assumed that sarcopenia may be associated with hearing impairment in middle-aged and elderly people.

Sarcopenia is a common and widespread case or physiological phenomenon characterized by a decrease in appendicular skeletal muscle mass (ASM), muscle strength and physical performance^17^. Globally, 10–27% of adults aged 60 years and older have sarcopenia^18^ and the prevalence of sarcopenia in people aged 80 years and older approaches 50%^19^. Sarcopenia has adverse effects on the physical and mental health of adults, such as falls, decreased function, cardiovascular disease, depression, cognitive impairment, death, and hospitalization^17,20–22^. Studies have found that adults with sarcopenia spend an average of $2315.7 more per year on medical care compared to adults without sarcopenia^23^. And the more severe the sarcopenia, the more money is spent on medical care^24^. There has been an ongoing debate about the diagnosis of sarcopenia. Diagnostic criteria for sarcopenia have been proposed by organizations such as the Asian Working Group on Sarcopenia (AWGS)^25^, the European Working Group on Sarcopenia in the Elderly (EWGSOP)^26^, the International Working Group on Sarcopenia (IWGS)^27^, and the Foundation for the National Institutes of Health (FNIH)^28^ in the U.S.A. In 2019, the AWGS has developed new diagnostic criteria for sarcopenia, based on AWGS 2014, and proposed “probable sarcopenia”, which provides a basis for diagnosis, prevention, and intervention of sarcopenia in middle-aged and elderly adults in community-based primary care and healthcare settings. According to AWGS 2019, low ASM and/or low muscle strength is diagnosed as probable sarcopenia, low ASM and low muscle strength or low physical performance is diagnosed as sarcopenia, and low ASM, low muscle strength, and low physical performance is diagnosed as severe sarcopenia^25^. Previous studies have found that both muscle strength and ASM have been associated with hearing impairments^29,30^. A cross-sectional study including 15,952 participants showed that sarcopenia has an effect on hearing loss^31^. Some studies have also inferred the effect of sarcopenia on hearing impairment from the perspective of physiological mechanisms^32,33^.

Currently, only two cross-sectional studies, a Mendelian randomized study and a prospective study, have reported the association between sarcopenia and hearing impairment, and there is no clear information about the effect of sarcopenia on hearing impairment in middle-aged and elderly adults. Therefore, this study investigated the association between sarcopenia and hearing impairment in middle-aged and elderly adults in China using longitudinal data from CHARLS 2011–2018 to provide a theoretical basis for preventing and delaying hearing impairment in middle-aged and elderly adults.

## Materials and methods

### Participants and procedures

CHARLS is a nationally representative longitudinal survey conducted in 28 provinces in China^34^. CHARLS pioneered the electronic mapping software (CHALRS-GIS) technology, used the map method to create village-level sampling frames, and adopted the PPS sampling method in multi-stage sampling, mainly surveying middle-aged and elderly people aged 45 years and above. More than 17,000 participants were surveyed at the baseline from 2011, and were followed up every 2 years by completing structured questionnaires mainly through one-on-one interviews. CHARLS questionnaires included personal information, household structure, economic support, health status, physical measurements, etc. CHARLS was approved by the Ethics Review Board of Peking University (No.: IRB00001052-11015) and was conducted in accordance with the Declaration of Helsinki and other relevant guidelines and regulations. All participants provided informed consent. This study follows the STROBE statement.

**Figure 1:**
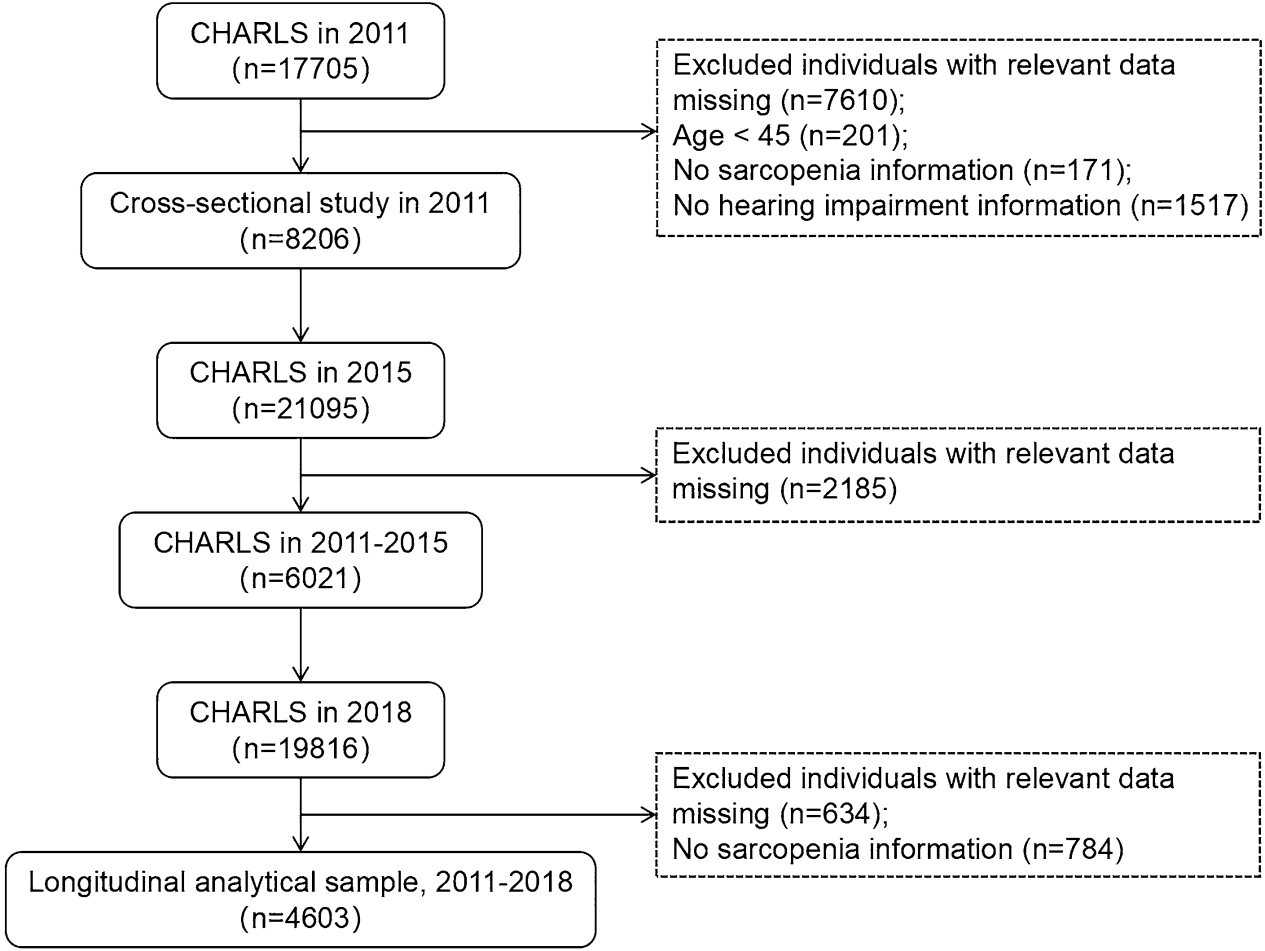
Flow diagram for participants included in the study.

Baseline 2011 and 2018 (Wave 4) data were used in this study. Inclusion criteria were (1) age ≥ 45 years in CHARLS 2011; and (2) availability of data on grip strength, ASM, gait speed, and five chair stand tests. Exclusion criteria were (1) age < 45 years in 2011; and (2) missing data on hearing condition. Multiple imputation was employed to handle the missing data on sarcopenia. Ultimately, based on the inclusion and exclusion criteria, there were a total of 9723 participants in 2011, with 8206 remaining after deleting participants with hearing impairments. In the longitudinal study, we further excluded participants with missing hearing condition data in 2018, resulting in a final residual of 4603 participants. The detailed participant selection process is shown in Fig. 1.

### Assessment of sarcopenia status

Sarcopenia was assessed according to the criteria recommended by AWGS 2019^25^ and consisted of three components: muscle strength, ASM and physical performance. Participants performed 2 grip strength measurements (kg) with their dominant and non-dominant hand. Participants were asked to stand with their elbows at a 90-degree angle to the side of the body and hold the Yuejian WL-1000 Grip Strength Device (Nantong Yuejian Physical Fitness Equipment Co.) with maximum force. The average of the maximum values of the two measurements of both hands was taken. The threshold values for assessing low grip strength in men and women were < 28 kg and < 18 kg, respectively. The study showed that the ASM equation modeling was very similar to the results of dual x-ray absorptiometry (DXA) for assessing human limb muscle mass. In this study, ASM in Chinese middle-aged and elderly people was calculated using measurement equations:

1 $$\begin{aligned} ASM = & 0.193 \times weight\;({\text{kg}}) + 0.107 \times height\;({\text{cm}}) \\ & \quad - 4.157 \times gender - 0.037 \times age\;({\text{years}}) - 2.631 \\ \end{aligned}$$

where height was measured using Seca™ 213 height meter, weight was measured using Omron™ HN-286 scale and gender was set to 1 (male) and 2 (female). The lowest 20% of ASM/Ht^2^ values according to gender is the threshold for low muscle mass^35,36^. In this study, ASM/Ht^2^ < 5.26 kg/m^2^ for females and ASM/Ht^2^ < 6.99 kg/m^2^ for males were considered as low ASM. Physical performance was assessed in this study using gait speed and five chair stand tests^35^. Each participant was asked to walk 2 times (back and forth) on a 2.5-m channel and the time for each time was recorded.

The five times chair stand test involved the participant interlocking their arms in front of their chest and sitting down straight from a 47 cm high chair five times as fast as they could without stopping or propping up with their arms in between. The total time taken and the number of times completed are recorded. Gait speed of < 1.0 m/s for a 6-m walk, or ≥ 12 s for 5 chair stand tests was considered low physical performance according to the AWGS 2019 criteria.

The participants in this study were divided into three groups: no sarcopenia (*n* = 956), possible sarcopenia (*n* = 3143), and sarcopenia (*n* = 504). “Possible sarcopenia” was defined as low ASM or low physical performance, while “sarcopenia” was defined as low ASM + low muscle strength or low ASM + low physical performance.

### Assessment of hearing impairment

Hearing impairment was assessed by participants’ self-reports, and studies have demonstrated the reliability of self-reported hearing status^37,38^. We used the CHARLS items “D*A038 Now I have some questions about your hearing. Do you ever wear a hearing aid?*” and “*DA039 Is your hearing very good, good, fair, poor, or very poor ?*” to test for hearing impairment. When DA038 selected “*Yes*” and/or DA039 selected “*Poor*”, the participant was considered to have a hearing impairment. Otherwise, the participant did not have a hearing impairment.

### Covariates

Demographic characteristics, lifestyle variables, physical and mental health indicators and metabolic biomarkers were included as covariates in this study. Demographic characteristics included age, sex, hukou status (birthplace or place of long-term residence), educational level (illiterate, elementary, junior high, high and middle school, college and bachelor’s degree and above), marital status (married, separated, divorced/widowed/unmarried), and annual income (< ¥1000, ¥1000, ¥5000, ¥10,000, ¥20,000). Lifestyle variables included hours of sleep at night, smoking, frequency of drinking (more than once a month, less than once a month, never). Physical and mental health indicators included body mass index (BMI), visual impairment, hypertension, dyslipidemia, diabetes, cancer, and depression. Metabolic biomarkers included mean corpuscular volume (MCV), total cholesterol (TC), triglycerides (TG), high density lipoprotein cholesterol (HDL-C), and low density lipoprotein cholesterol (LDL-C). Among them, participants were categorized into overweight and obese group (BMI ≥ 24 kg/m^2^), normal weight group (18.5 kg/m^2^ ≤ BMI < 24 kg/m^2^) and underweight group (BMI < 18.5 kg/m^2^) based on BMI. Depression was scored according to the 10-item Center for Epidemiologic Studies Depression Scale (CESD-10) with a total score of 30 (≥ 10 being depressed).

### Statistical analyses

This study contains mainly cross-sectional and longitudinal analyses. We used mean ± standard deviation to describe continuous variables and number and percentage to describe categorical variables. First, according to the category of sarcopenia, comparative analyses were performed using chi-square test and ANOVA to characterize the baseline data in 2011 and the longitudinal data from 2011 to 2018, respectively. Second, binary logistic regression analyses were used to estimate the associations of possible sarcopenia and sarcopenia with hearing impairment in the 2011 cross-sectional data, as indicated by regression coefficients (β), odds ratios (ORs), and 95% confidence interval (95%CI). Then, in longitudinal analyses, we measured follow-up time and used cox proportional risk models to assess the association between baseline sarcopenia status and hearing impairment, expressed using hazard ratios (HR) and 95%CI.

We estimated three models in both cross-sectional and longitudinal analyses, and the three models used different combinations of covariates. Model 1 included age and gender; model 2 included age, gender, marital status, hukou status, educational level, smoking, frequency of drinking, hours of sleep at night and annual income; and model 3 added BMI, visual impairment, hypertension, dyslipidemia, diabetes mellitus, cancer, depression, MCV, TC, TG, HDL-C and LDL-C to model 2. All statistical analyses were performed using the SPSS 26.0 and R version 4.3.1 and were considered statistically significant when *p* < 0.05.

### Ethical approval

CHARLS was approved by the Ethics Review Committee of Peking University (No. IRB00001052-11015).

## Results

### Basic characteristics of participants

Table S1 in the Appendix shows the baseline characteristics of all participants categorized according to sarcopenia status. The mean age of the participants was 59.59 (± 9.43) years, 5205 (53.5%) were female, and 8206 (84.4%) participants had hearing impairment. The prevalence of hearing impairment was 15.14% for participants without sarcopenia, 13.80% for probable sarcopenia, and 24.04% for sarcopenia. Among participants with possible sarcopenia and sarcopenia, there was a higher proportion of females, those with a rural household registration, lower education levels, married individuals, low incomes, non-smokers, non-drinkers, and those with visual impairments (all *p* < 0.001). Table S2 in the Appendix shows the baseline characteristics of participants without hearing impairment (*n* = 8206).

### Cross-sectional association of probable sarcopenia and sarcopenia with hearing impairment

**Figure 2:**
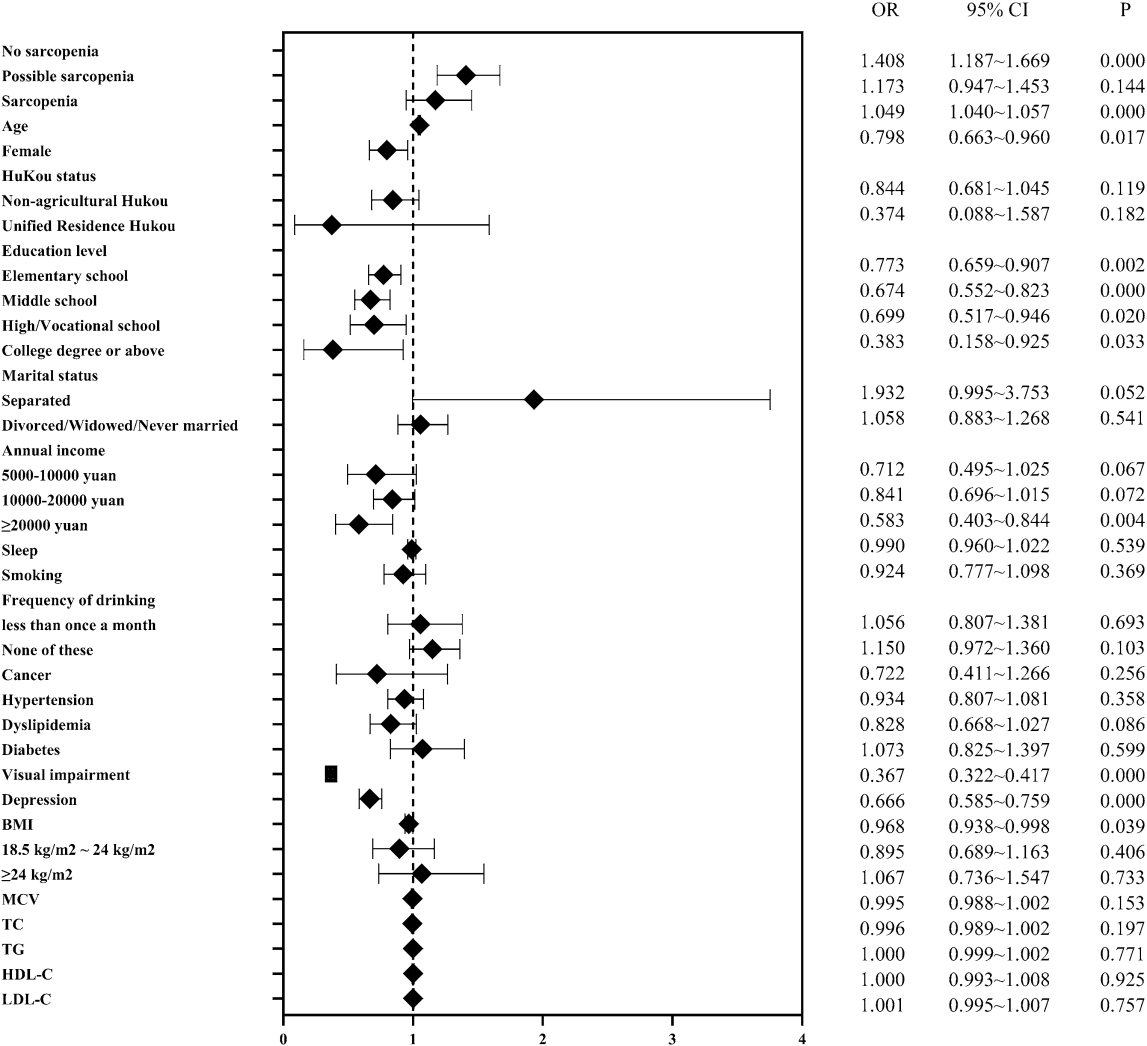
ORs and 95% CIs of hearing impairment by sarcopenia status in the cross-sectional analysis. The forest plot shows the ORs and 95% CIs for Model 3, which adjusts for age, gender, marital status, hukou status, educational level, smoking, frequency of drinking, hours of sleep at night, annual income, BMI, visual impairment, hypertension, dyslipidemia, diabetes mellitus, cancer, depression, MCV, TC, TG, HDL-C and LDL-C.

In cross-sectional analyses, we used binary logistic regression to analyze the association of probable sarcopenia and sarcopenia with hearing impairment. After adjusting for demographic characteristics and lifestyle variables, both probable sarcopenia and sarcopenia were significantly and positively associated with hearing impairment (*p* < 0.01). On this basis, after adjusting for physical and mental health indicators and metabolic biomarkers, probable sarcopenia was significantly and positively associated with hearing impairment [0.342 (1.187, 1.669), *p* < 0.001], but sarcopenia was not significantly associated with hearing impairment [0.159 (0.947, 1.453), *p* = 0.144] (Table 1). Figure 2 shows the ORs and 95% CIs of sarcopenia status and covariates for hearing impairment in model 3.

**Table 1:** Binary logistic regression model of sarcopenia and hearing impairment.

Outcome	Cases, n (%)	OR (95% CI)		
		Model 1	Model 2	Model 3
Hearing impairment				
No sarcopenia (n = 1935)	293 (15.14%)	Reference	Reference	Reference
Possible sarcopenia (n = 6332)	874 (13.80%)	0.395 (1.264,1.742)***	0.370 (1.230,1.702)***	0.342 (1.187,1.669)***
Sarcopenia (n = 1456)	350 (24.04%)	0.448 (1.296,1.890)***	0.334 (1.153,1.690)**	0.159 (0.947,1.453)

OR, odds ratio. *p < 0.05, **p < 0.01, ***p < 0.001.

Model 1 was adjusted for age and gender.

Model 2 was adjusted for age, gender, marital status, hukou status, educational level, smoking, frequency of drinking, hours of sleep at night and annual income.

Model 3 builds on and further adjusts for BMI, visual impairment, hypertension, dyslipidemia, diabetes mellitus, cancer, depression, MCV, TC, TG, HDL-C and LDL-C in model 2.

### Longitudinal association of probable sarcopenia and sarcopenia with hearing impairment

**Figure 3:**
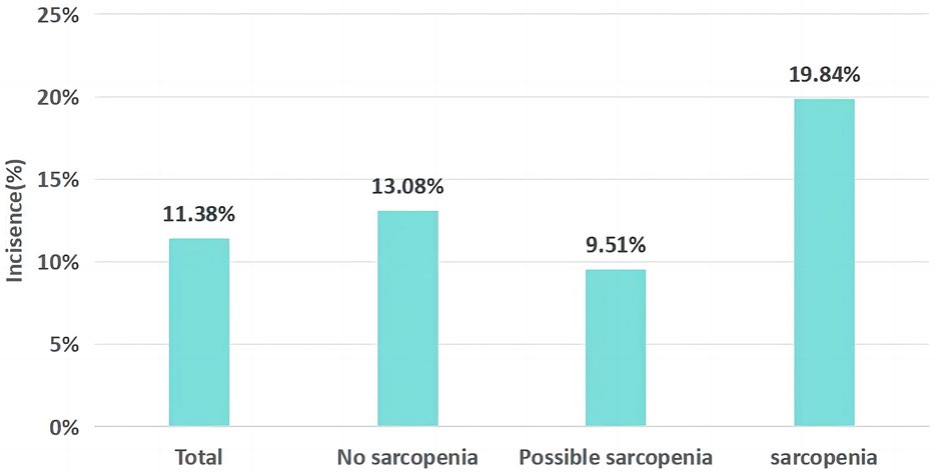
Prevalence of hearing impairment in different groups.

**Figure 4:**
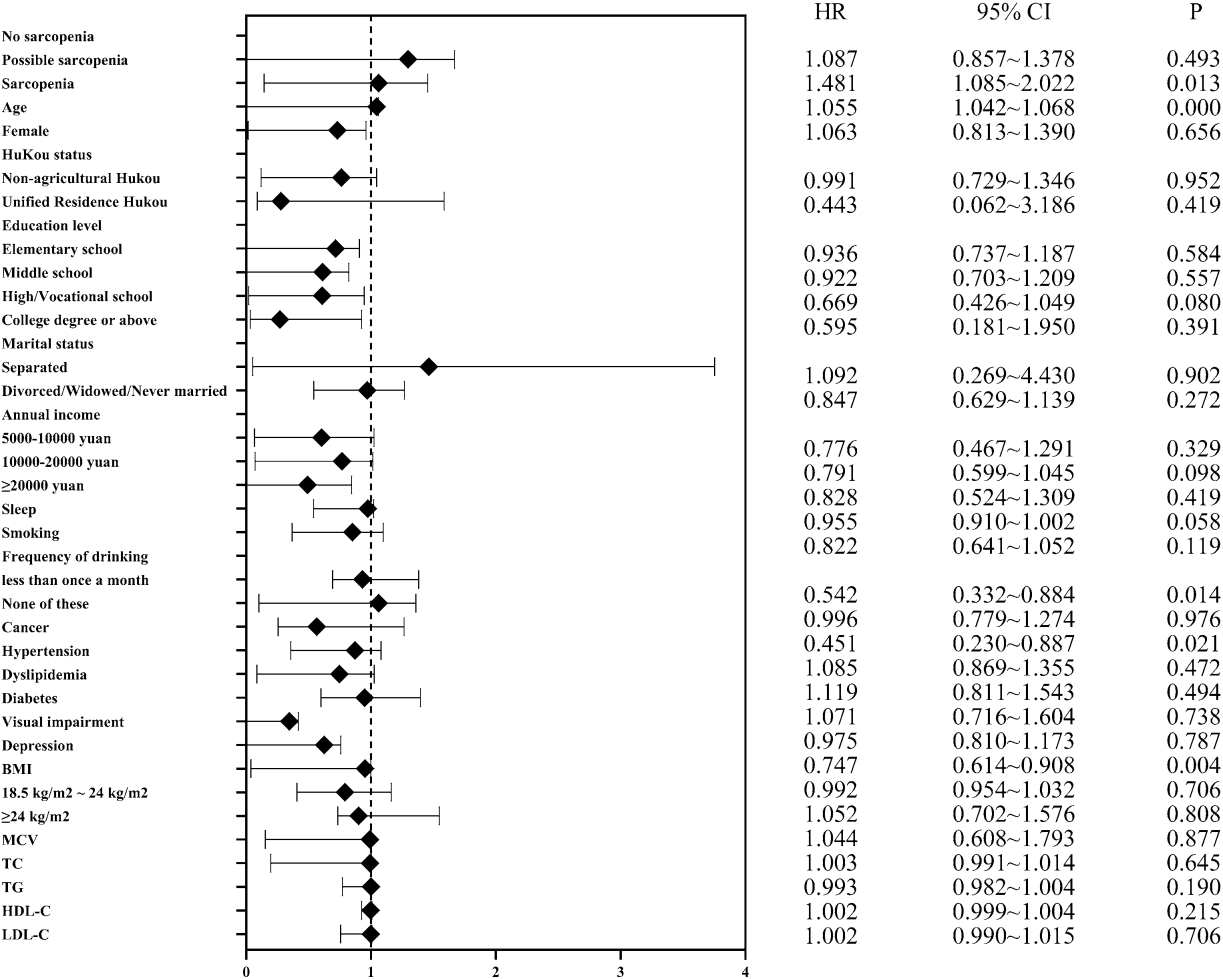
Longitudinal association of baseline sarcopenia status with hearing impairment, 2011–2018. The forest plot shows the hazard ratios (HRs) and 95% CIs for Model 3, which adjusts for age, gender, marital status, hukou status, educational level, smoking, frequency of drinking, hours of sleep at night, annual income, BMI, visual impairment, hypertension, dyslipidemia, diabetes mellitus, cancer, depression, MCV, TC, TG, HDL-C and LDL-C.

In a longitudinal analysis of 4603 participants, 524 middle-aged and elderly adults had hearing impairment in 2018. The prevalence of hearing impairment was 13.08% in no sarcopenia, 9.51% in probable sarcopenia, and 19.84% in sarcopenia (see Fig. 3). Table 2 shows the longitudinal associations of probable sarcopenia and sarcopenia with hearing impairment. After adjusting for demographic characteristics, participants with sarcopenia were more likely to have hearing impairment than participants without sarcopenia and probable sarcopenia [0.447 (1.189, 2.057), *p* < 0.01]. Participants with probable sarcopenia were not significantly associated with an increased risk of hearing impairment compared to participants without sarcopenia. After sequential adjustment for lifestyle variables, physical and mental health indicators and metabolic biomarkers, the longitudinal associations between sarcopenia and hearing impairment remained significant [0.411 (1.143, 1.990), *p* < 0.01], [0.354 (1.043, 1.945), *p* < 0.01], and the longitudinal associations between probable sarcopenia and hearing impairment remained nonsignificant. Figure 4 shows the HRs and 95% CIs of sarcopenia status and covariates for hearing impairment in model 3.

**Table 2:** Incidence of hearing impairment according to baseline sarcopenia status, 2011–2018.

Outcome	Cases, n (%)	HR (95% CI)		
		Model 1	Model 2	Model 3
Hearing impairment				
No sarcopenia (n = 956)	125 (13.08%)	Reference	Reference	Reference
Possible sarcopenia (n = 3143)	299 (9.51%)	0.101 (0.876,1.398)	0.093 (0.868,1.388)	0.073 (0.849,1.365)
Sarcopenia (n = 504)	100 (19.84%)	0.447 (1.189,2.057)**	0.411 (1.143,1.990)**	0.354 (1.043,1.945)**

HR, hazard ratio. *p < 0.05, **p < 0.01, ***p < 0.001.

Model 1 was adjusted for age and gender.

Model 2 was adjusted for age, gender, marital status, hukou status, educational level, smoking, frequency of drinking, hours of sleep at night and annual income.

Model 3 builds on and further adjusts for BMI, visual impairment, hypertension, dyslipidemia, diabetes mellitus, cancer, depression, MCV, TC, TG, HDL-C and LDL-C in model 2.

## Discussion

The purpose of this study was to investigate the impact of sarcopenia on hearing impairment in middle-aged and elderly adults in China. It is understood that this is the first study to examine the longitudinal relationship between sarcopenia and hearing impairment in a middle-aged and elderly population using a nationally representative sample. Results of cross-sectional analyses showed that both middle-aged and elderly adults with probable sarcopenia and sarcopenia were significantly positively associated with hearing impairment after adjusting for demographic characteristics and lifestyle variables. After adjusting for physical and mental health indicators and metabolic biomarkers, only the probable sarcopenia was significantly positively associated with hearing impairment. Longitudinal analyses showed that middle-aged and elderly adults with sarcopenia had a higher risk of hearing impairment than middle-aged and elderly adults without sarcopenia and probable sarcopenia, after adjusting for all covariates.

The results of our study are not entirely consistent with those of previous cross-sectional studies^30^. A study targeting Korean elders aged 60 and over showed that older female adults with greater muscle mass had better hearing, whereas there was no significant connection between muscle mass and hearing in male elders^30^. Furthermore, another study revealed that older adult women with sarcopenia had a higher incidence of hearing impairment compared to those without sarcopenia^39^. A Mendelian randomization study found a significant relationship between walking speed, ASM, and grip strength with hearing impairment, and a causal relationship between skeletal muscle mass and hearing impairment in women^31^. The previous longitudinal study with a follow-up of 5.8 years found a significant negative correlation between muscle mass and BMI with hearing impairment in middle-aged and elderly adults^29^, revealing the potential link between sarcopenia and hearing impairment. In regards to the inconsistency between the findings of this study and previous studies, we believe that, on one hand, it could be due to the different diagnostic criteria for “sarcopenia” compared to previous research. The diagnostic criteria for “sarcopenia” in this study were based on the latest version of AWGS 2019, categorizing participants into “no sarcopenia”, “possible sarcopenia”, and “sarcopenia”^25^. Currently, there is no existing literature using AWGS 2019 to investigate the association between possible sarcopenia, sarcopenia, and hearing impairment in middle-aged and elderly adults. On the other hand, this may be predominantly attributed to women experiencing long-term estrogen deficiency after menopause, which may increase the risk of hypertension, cardiovascular diseases, osteoporosis, and the likelihood of developing sarcopenia^40^, ultimately having a more pronounced impact on auditory function. Additionally, we found that after adjusting for physical and mental health indicators and metabolic biomarkers, the relationship between sarcopenia and hearing impairment was not significant, while the relationship between possible sarcopenia and hearing impairment remained evident. We speculate that some factors within the physical health indicators and metabolic biomarkers may have a more significant influence on hearing impairment, potentially mediating or moderating the association between sarcopenia and hearing impairment. Therefore, future research could further explore this direction.

In the longitudinal analysis, the longitudinal association between sarcopenia and hearing impairment was significantly correlated compared with middle-aged and elderly adults without sarcopenia, and probable sarcopenia and hearing impairment was not significantly correlated. The results are consistent with those of previous studies^29,30^. Studies in both Korea and Japan have found that sarcopenia increases the risk of hearing loss in older adults, especially in women^29,30^. This may be due to the fact that, on the one hand, skeletal muscle is able to take up, oxidize, and store fatty acids to participate in lipid metabolism, thereby maintaining the balance of lipid metabolism in the body^41^. Sarcopenia leads to metabolic disorders, which can cause hyperlipidemia, atherosclerosis, peripheral vascular disease and stroke^41^. Chronic hyperlipidemia can lead to increased blood viscosity, narrowing of blood vessels, and the formation of microthrombi. More skeletal muscle requires more blood supply, more arterial blood flow and larger arterial diameter^42,43^. Sarcopenia results in insufficient arterial blood flow and smaller arterial diameter, which leads to reduced blood flow to the cochlea, making hearing function reduced^44^. On the other hand, cochlear ischemia is associated with damage to the vascular stripe, which leads to hearing impairment^45^^.^ Metabolic disorders can lead to diabetes^46^, and in turn, diabetes can exacerbate metabolic disorders and microangiopathy. The most common of these are microvascular lesions in the cochlear vascular stripe^33^. These lesions cause degeneration of the cochlea and cochlear nerve, which can lead to hearing loss^47^. In addition, endothelial dysfunction caused by diabetes and hyperglycemia leads to atrophy or damage of the cells of the vascular stripe, which results in disruption of the ion transport system in the vascular stripe and abnormalities in the connecting proteins of the gap junctions, leading to disruption of the formation of the electrical potentials in the cochlea, and to the occurrence of hearing impairment^48,49^. This study helps to increase the awareness of middle-aged and elderly adults regarding sarcopenia and hearing impairment, allowing family members to pay closer attention to and take care of their relatives’ hearing health in advance. It also helps communities and medical institutions to timely diagnose, prevent, and intervene in sarcopenia in middle-aged and elderly adults, develop new prevention strategies and treatment plans, and reduce and delay the occurrence of hearing impairment. Additionally, this study provides policy makers with an understanding of the latest research progress in hearing impairment, allowing them to formulate more comprehensive hearing health policies.

### Strengths and limitations

Our research results not only support the effectiveness of the AWGS 2019 diagnostic algorithm for sarcopenia in Asian older adults but also provide new evidence for the causal relationship between probable sarcopenia, sarcopenia and hearing impairment. The strengths of this study are reflected in the sample selection and prospective study. First, this study used the CHARLS database, which is a nationally representative survey data that can represent the overall level of middle-aged and elderly people in China. Therefore, the study results are more realistic and reliable. Second, our study used longitudinal data with 7 years of follow-up and was able to reveal the causal relationship between sarcopenia and hearing impairment in middle-aged and elderly adults, making it the first study to explore the longitudinal relationship between sarcopenia and possible sarcopenia and hearing impairment in middle-aged and elderly adults in China.

There are also several limitations to this study. First, we used observational data, which may have biased the relationships between variables due to other confounding variables. Therefore, we adjusted for other potential confounders as much as possible, including 21 covariates for demographic characteristics, lifestyle variables, physical and mental health indicators, and metabolic biomarkers. Second, the diagnostic criteria regarding hearing impairment was through participants’ self-report, and no diagnostic records were available in CHARLS. Therefore, there may be some bias in the study results. In addition, the grading and types of hearing impairment were not addressed in CHARLS. Therefore, we could not analyze the relationship between sarcopenia and hearing impairment in depth.

## Conclusions

In conclusion, this study evaluated probable sarcopenia and sarcopenia in middle-aged and elderly adults in China using the AWGS 2019 diagnostic criteria and explored the association and impact of probable sarcopenia and sarcopenia with hearing impairment, and found that there was a significant association between probable sarcopenia and hearing impairment, and that sarcopenia increased the risk of developing hearing impairment.

### Supplementary Information

Supplementary Tables.

## Data Availability

The raw data for this study is available from: http://charls.pku.edu.cn/en. The dataset generated from this study is available from the corresponding author.
