# Supplementary material for: Association between sarcopenia and hearing impairment in middle-aged and elderly people in China: a prospective cohort study
Source: Sci Rep. 2024 Mar 13;14:6061. doi: 10.1038/s41598-024-56850-4 (PMC10937624; doi:10.1038/s41598-024-56850-4)
Supplement: Supplementary file 1 — Supplementary Tables. [file 41598_2024_56850_MOESM1_ESM.docx]

**Supplementary material**

**Association between sarcopenia and hearing impairment in middle-aged and** **elderly people** **in China: a prospective cohort study**

**Table S1.** Baseline characteristics of all participants by sarcopenia status.

| **Characteristics** | **Total** | **No sarcopenia** | **Possible sarcopenia** | **Sarcopenia** | **p** |
| --- | --- | --- | --- | --- | --- |
| Hearing impairment, n(%) |  |  |  |  | <0.001 |
| Yes | 1517(15.60) | 293(15.14) | 874(13.80) | 350(24.04) |  |
| No | 8206(84.40) | 1642(84.86) | 5458(86.20) | 1106(75.96) |  |
| Age, year (mean±SD) | 59.59±9.43 | 65.81±5.15 | 56.19±8.36 | 66.12±10.48 | <0.001 |
| Gender, n(%) |  |  |  |  | <0.001 |
| Male | 4518(46.50) | 1094(56.54) | 2792(44.09) | 632(43.41) |  |
| Female | 5205(53.50) | 841(43.46) | 3540(55.91) | 824(56.59) |  |
| Hukou status, n(%) |  |  |  |  | <0.001 |
| Agricultual | 8030(82.60) | 1509(77.98) | 5207(82.27) | 1314(90.25) |  |
| Non-agricultural | 1646(16.90) | 416(21.50) | 1090(17.22) | 140(9.62) |  |
| Unified Residence | 43(0.40) | 10(0.52) | 31(0.49) | 2(0.14) |  |
| Do not have Hukou | 1(0.00) | 0(0.00) | 1(0.02) | 0(0.00) |  |
| Educational level, n(%) |  |  |  |  | <0.001 |
| Illiterate | 4646(47.80) | 948(48.99) | 2732(43.15) | 966(66.44) |  |
| Elementary | 2208(22.70) | 558(28.84) | 1365(21.56) | 285(19.60) |  |
| Junior high | 1921(19.80) | 282(14.57) | 1491(23.55) | 148(10.18) |  |
| High and middle school | 814(8.40) | 107(5.53) | 656(10.36) | 51(3.51) |  |
| College and bachelor's degree and above | 131(1.30) | 40(2.07) | 87(1.37) | 4(0.28) |  |
| Marital status, n(%) |  |  |  |  | <0.001 |
| Married | 8481(87.20) | 1654(85.48) | 5721(90.35) | 1106(75.96) |  |
| Separated | 52(0.50) | 11(0.57) | 28(0.44) | 13(0.89) |  |
| Divorced/widowed/unmarried | 1190(12.20) | 270(13.95) | 583(9.21) | 337(23.15) |  |
| Annual income, n(%) |  |  |  |  | <0.001 |
| <1,000 | 7182(73.90) | 1336(69.04) | 4660(73.64) | 1186(81.51) |  |
| 1,000-5000 | 388(4.00) | 73(3.77) | 290(4.58) | 25(1.72) |  |
| 5000-10000 | 1457(15.00) | 339(17.52) | 910(14.38) | 208(14.30) |  |
| 10000-20000 | 691(7.10) | 187(9.66) | 468(7.40) | 36(2.47) |  |
| Smoking, n(%) |  |  |  |  | <0.001 |
| Yes | 3840(39.50) | 913(47.18) | 2329(36.79) | 598(41.07) |  |
| No | 5882(60.50) | 1022(52.82) | 4002(63.21) | 858(58.93) |  |
| Frequency of drinking, n(%) |  |  |  |  | <0.001 |
| More than once a month | 2430(25.00) | 570(29.46) | 1523(24.05) | 337(23.15) |  |
| Less than once a month | 752(7.70) | 155(8.01) | 511(8.07) | 86(5.91) |  |
| Never | 6541(67.30) | 1210(62.53) | 4298(67.88) | 1033(70.95) |  |
| Hours of sleep at night (mean±SD) | 6.343±1.89 | 6.30±1.84 | 6.42±1.83 | 6.06±2.19 | <0.001 |
| BMI, kg/m^2^ (mean±SD) | 23.53±3.93 | 23.24±3.77 | 24.65±3.54 | 18.97±1.82 | <0.001 |
| BMI<18.5 | 665(6.90) | 137(7.09) | 1(0.02) | 527(36.85) |  |
| 18.5≤BMI<24 | 5049(52.10) | 1040(53.83) | 3113(49.16) | 896(62.66) |  |
| BMI≥24 | 3980(41.10) | 755(39.08) | 3218(50.82) | 7(0.49) |  |
| Visual impairment, n(%) |  |  |  |  | <0.001 |
| Yes | 4167(42.90) | 835(43.15) | 2616(41.31) | 716(49.18) |  |
| No | 5556(57.10) | 1100(56.85) | 3716(58.69) | 740(50.82) |  |
| Hypertension, n(%) |  |  |  |  | <0.001 |
| Yes | 2445(25.30) | 570(29.55) | 1617(25.72) | 258(17.84) |  |
| No | 7217(74.70) | 1359(70.45) | 4670(74.28) | 1188(82.16) |  |
| Dyslipidemia, n(%) |  |  |  |  | <0.001 |
| Yes | 902(9.50) | 208(10.89) | 642(10.41) | 52(3.67) |  |
| No | 8592(90.50) | 1702(89.11) | 5527(89.59) | 1363(96.33) |  |
| Diabetes, n(%) |  |  |  |  | <0.001 |
| Yes | 557(5.80) | 119(6.19) | 396(6.33) | 42(2.92) |  |
| No | 9061(94.20) | 1803(93.81) | 5862(93.67) | 1396(97.08) |  |
| Cancer, n(%) |  |  |  |  | 0.306 |
| Yes | 95(1.00) | 13(0.67) | 66(1.04) | 16(1.10) |  |
| No | 9628(99.00) | 1922(99.33) | 6266(98.96) | 1440(98.90) |  |
| Depression, n(%) |  |  |  |  | <0.001 |
| Yes | 3662(37.70) | 638(32.97) | 2300(36.32) | 724(49.73) |  |
| No | 6061(62.30) | 1297(67.03) | 4032(63.68) | 732(50.27) |  |
| MCV, fl (mean±SD) | 90.69±8.57 | 91.09±8.53 | 90.35±8.43 | 91.65±9.11 | <0.001 |
| TC, mg/dL (mean±SD) | 193.88±38.96 | 195.24±38.82 | 194.30±39.29 | 190.23±37.49 | <0.001 |
| TG, mg/dL (mean±SD) | 133.99±110.45 | 127.83±95.26 | 142.92±121.90 | 103.40±58.93 | <0.001 |
| HDL-C, mg/dL (mean±SD) | 51.13±15.33 | 51.67±15.82 | 49.39±14.49 | 57.96±16.26 | <0.001 |
| LDL-C, mg/dL (mean±SD) | 116.63±35.09 | 119.09±35.67 | 116.74±35.26 | 112.88±33.20 | <0.001 |

Abbreviation: body mass index, BMI; mean corpuscular volume, MCV; total cholesterol, TC; triglycerides, TG; high density lipoprotein cholesterol, HDL-C; low density lipoprotein cholesterol, LDL-C.

**Table S2.** Baseline characteristics of participants without hearing impairment by sarcopenia status (n = 8206).

| **Characteristics** | **Total** | **No sarcopenia** | **Possible sarcopenia** | **Sarcopenia** | **p** |
| --- | --- | --- | --- | --- | --- |
| Age, year (mean±SD) | 58.77±9.04 | 65.59±4.92 | 55.48±7.83 | 64.92±10.33 | <0.001 |
| Gender, n(%) |  |  |  |  | <0.001 |
| Male | 3800(46.31) | 65.59±4.92 | 55.48±7.83 | 64.92±10.33 |  |
| Female | 4406(53.69) | 65.59±4.92 | 55.48±7.83 | 64.92±10.33 |  |
| Hukou status, n(%) |  |  |  |  | <0.001 |
| Agricultual | 6689(81.54) | 1260(76.74) | 4441(81.41) | 988(89.33) |  |
| Non-agricultural | 1472(17.94) | 373(22.72) | 983(18.02) | 116(10.49) |  |
| Unified Residence | 41(0.50) | 9(0.55) | 30(0.55) | 2(0.18) |  |
| Do not have Hukou | 1(0.01) | 0(0.00) | 1(0.02) | 0(0.00) |  |
| Educational level, n(%) |  |  |  |  | <0.001 |
| Illiterate | 3693(45.00) | 779(47.44) | 2213(40.55) | 701(63.38) |  |
| Elementary | 1896(23.11) | 479(29.17) | 1189(21.78) | 228(20.61) |  |
| Junior high | 1744(21.25) | 251(15.29) | 1363(24.97) | 130(11.75) |  |
| High and middle school | 750(9.14) | 95(5.79) | 611(11.19) | 44(3.98) |  |
| College and bachelor's degree and above | 123(1.50) | 38(2.31) | 82(1.50) | 3(0.27) |  |
| Marital status, n(%) |  |  |  |  | <0.001 |
| Married | 7270(88.59) | 1415(86.18) | 4989(91.41) | 866(78.30) |  |
| Separated | 36(0.44) | 6(0.37) | 20(0.37) | 10(0.90) |  |
| Divorced/widowed/unmarried | 900(10.97) | 221(13.46) | 449(8.23) | 230(20.80) |  |
| Annual income, n(%) |  |  |  |  | <0.001 |
| <1,000 | 5954(72.60) | 1109(67.54) | 3952(72.46) | 893(80.81) |  |
| 1,000-5000 | 350(4.27) | 65(3.96) | 263(4.82) | 22(1.99) |  |
| 5000-10000 | 1253(15.28) | 292(17.78) | 801(14.69) | 160(14.48) |  |
| 10000-20000 | 644(7.85) | 176(10.72) | 438(8.03) | 30(2.71) |  |
| Smoking, n(%) |  |  |  |  | <0.001 |
| Yes | 3213(39.16) | 774(47.14) | 1996(36.58) | 443(40.05) |  |
| No | 4992(60.84) | 868(52.86) | 3461(63.42) | 663(59.95) |  |
| Frequency of drinking, n(%) |  |  |  |  | <0.001 |
| More than once a month | 2084(25.40) | 489(29.78) | 1341(24.57) | 254(22.97) |  |
| Less than once a month | 653(7.96) | 134(8.16) | 457(8.37) | 62(5.61) |  |
| Never | 5469(66.65) | 1019(62.06) | 3660(67.06) | 790(71.43) |  |
| Hours of sleep at night (mean±SD) | 6.40±1.84 | 6.32±1.80 | 6.48±1.79 | 6.12±2.12 | <0.001 |
| BMI, kg/m^2^ (mean±SD) | 23.65±3.93 | 23.41±3.76 | 24.66±3.58 | 18.94±1.83 | <0.001 |
| BMI<18.5 | 511(6.24) | 103(6.28) | 1(0.02) | 407(37.48) |  |
| 18.5≤BMI<24 | 4229(51.68) | 870(53.08) | 2687(49.23) | 672(61.88) |  |
| BMI≥24 | 3443(42.08) | 666(40.63) | 2770(50.75) | 7(0.64) |  |
| Visual impairment, n(%) |  |  |  |  | 0.010 |
| Yes | 3168(38.61) | 648(39.46) | 2052(37.60) | 468(42.31) |  |
| No | 5038(61.39) | 994(60.54) | 3406(62.40) | 638(57.69) |  |
| Hypertension, n(%) |  |  |  |  | <0.001 |
| Yes | 2011(24.65) | 481(29.40) | 1345(24.79) | 185(16.85) |  |
| No | 6148(75.35) | 1155(70.60) | 4080(75.21) | 913(83.15) |  |
| Dyslipidemia, n(%) |  |  |  |  | <0.001 |
| Yes | 753(9.39) | 171(10.54) | 542(10.18) | 40(3.73) |  |
| No | 7269(90.61) | 1452(89.46) | 4784(89.82) | 1033(96.27) |  |
| Diabetes, n(%) |  |  |  |  | <0.001 |
| Yes | 468(5.76) | 104(6.36) | 331(6.13) | 33(3.03) |  |
| No | 7652(94.24) | 1530(93.64) | 5065(93.87) | 1057(96.97) |  |
| Cancer, n(%) |  |  |  |  | 0.651 |
| Yes | 76(0.93) | 12(0.73) | 53(0.97) | 11(0.99) |  |
| No | 8130(99.07) | 1630(99.27) | 5405(99.03) | 1095(99.01) |  |
| Depression, n(%) |  |  |  |  | <0.001 |
| Yes | 2872(35.00) | 507(30.88) | 1843(33.77) | 522(47.20) |  |
| No | 5334(65.00) | 1135(69.12) | 3615(66.23) | 584(52.80) |  |
| MCV, fl (mean±SD) | 90.67±8.53 | 91.17±8.44 | 90.36±8.43 | 91.46±9.07 | <0.001 |
| TC, mg/dL (mean±SD) | 194.38±39.23 | 195.84±38.82 | 194.70±39.64 | 190.64±37.62 | 0.002 |
| TG, mg/dL (mean±SD) | 134.81±110.65 | 130.09±98.96 | 142.58±120.25 | 103.60±60.60 | <0.001 |
| HDL-C, mg/dL (mean±SD) | 51.10±15.33 | 51.51±15.62 | 49.47±14.57 | 58.56±16.30 | <0.001 |
| LDL-C, mg/dL (mean±SD) | 116.93±35.37 | 119.39±35.53 | 117.07±35.65 | 112.58±33.29 | <0.001 |

Abbreviation: body mass index, BMI; mean corpuscular volume, MCV; total cholesterol, TC; triglycerides, TG; high density lipoprotein cholesterol, HDL-C; low density lipoprotein cholesterol, LDL-C.
